# Association of Conicity Index and Body Roundness Index with Multimorbidity Among Adults in Guangzhou, China: A Cross-Sectional Study and Implications for Nutritional Risk Stratification

**DOI:** 10.3390/nu18142286

**Published:** 2026-07-13

**Authors:** Jiamin Chen, Yingying Fang, Weiquan Lin, Liying Luo, Minying Sun, Yaohui Li, Lan Liu, Yunou Yang, Lei Shi, Hui Liu

**Affiliations:** 1 Department of Basic Public Health, Guangzhou Center for Disease Control and Prevention (Guangzhou Health Supervision Institute), Guangzhou 510440, China; gzcdc_chenjm@gz.gov.cn (J.C.); gzcdc_fangyy@gz.gov.cn (Y.F.); linweiquan0503@163.com (W.L.); luoliying_0928@163.com (L.L.); gzcdc_liyh@gz.gov.cn (Y.L.); gzcdc_yangyo@gz.gov.cn (Y.Y.); 2 Institute of Public Health, Guangzhou Medical University & Guangzhou Center for Disease Control and Prevention (Guangzhou Health Supervision Institute), Guangzhou 510440, China; sunmy1220@163.com (M.S.); gzcdc_liul@gz.gov.cn (L.L.); 3 Department of Science and Education Information, Guangzhou Center for Disease Control and Prevention (Guangzhou Health Supervision Institute), Guangzhou 510440, China; 4 Department of Foodborne Diseases and Food Safety Risk Surveillance, Guangzhou Center for Disease Control and Prevention (Guangzhou Health Supervision Institute), Guangzhou 510440, China; 5 School of Health Management, Guangzhou Medical University, Guangzhou 510182, China; 6 Social Science Key Laboratory of Guangdong Higher Education Institutes for Health Management Policy and Precision Health Services, Guangzhou 510515, China; 7 Social Science Key Laboratory of Guangdong Higher Education Institutes for Health Governance Based on Big Data Utilization, Guangzhou 511436, China

**Keywords:** multimorbidity, conicity index, body roundness index, abdominal obesity, logistic regression, nutritional risk stratification

## Abstract

Background: Optimizing nutritional care for people with multimorbidity requires effective and feasible tools for early risk identification. This study aimed to investigate the associations of conicity index (C-index) and body roundness index (BRI) with multimorbidity among adults in Guangzhou, and to explore their potential utility in identifying individuals who may benefit from prioritized nutritional assessment. Methods: Based on the Fourth Adult Chronic Disease and Risk Factor Surveillance Program in Guangzhou, a total of 15,532 adults were recruited in this cross-sectional study. Multiple logistic regression analysis, restricted cubic spline (RCS) analysis and receiver operating characteristic curve (ROC) analysis with DeLong’s test were performed. Results: The prevalence of multimorbidity was 38.95%. Higher C-index and BRI quartiles were significantly associated with increased multimorbidity risk. Compared with the lowest quartile, the adjusted OR for the highest C-index quartile was 1.98 (95% CI: 1.78–2.21), and for the highest BRI quartile it was 3.05 (95% CI: 2.74–3.41). A dose–response relationship was observed (*p* < 0.001). BRI showed statistically significantly higher discriminative ability relative to traditional anthropometric indices, though its overall discriminative performance was modest (AUC = 0.678). Conclusions: C-index and BRI are strongly and positively associated with multimorbidity. These simple, non-invasive indices may support community-based risk stratification and help identify individuals who warrant further clinical and nutritional assessment.

## 1. Introduction

Multimorbidity is most commonly defined as the co-existence of two or more chronic conditions within an individual [1]. A study showed the overall global prevalence of multimorbidity was 37.2%, and that of Asia was 35% [2]. Moreover, a systematic review and meta-analysis revealed the prevalence of multimorbidity was 25.4% among Chinese adults [3]. Multimorbidity increased medical consultations and hospitalizations [4,5], elevated the risk of disability [6,7], depression [8], complications [9], and death [10,11,12]. People suffering from multimorbidity were more likely to live with poor physical function and quality of life [13], which had negative impacts on the household economic burden and the utilization of social health resources. As the prevalence of multimorbidity rises with the growth of age [14], prevention and health management of multimorbidity are particularly important with the globally rapid aging trend today. Optimizing nutritional care, including nutritional screening, dietary assessment, personalized counseling, and weight management, is a key strategy in multimorbidity management. Recent evidence indicates that nutritional support can reduce morbidity and other complications in patients with multimorbidity [15]. However, delivering effective nutritional care at scale requires feasible tools to identify high-risk individuals who need prioritized clinical and nutritional assessment and even further intervention. Currently, stratification tools for assessing individuals with multimorbidity are rare, and existing instruments vary considerably in their scoring methods and settings of use. Moreover, few simple, non-invasive screening tools are available for use in community or primary care settings [16].

Identifying risk factors is the first step in developing screening tools. Among these factors, obesity stands out as both a strong contributor to multimorbidity and a measurable target for nutritional intervention [17,18]. Obesity has become a global public health issue [19,20], with its prevalence markedly increasing worldwide and nearly half of Chinese adults now being overweight or obese [19,21]. Although body mass index (BMI) is commonly used to measure obesity [17], it does not effectively distinguish muscle from fat nor reflect body fat distribution [22]. Evidence suggested that, compared with overall obesity, body fat distribution, especially the accumulation of abdominal adiposity, has a substantial impact on health and is more closely associated with disease development [23].

Waist circumference (WC) and waist-to-hip ratio (WHR) are commonly used to assess central obesity and have been associated with multimorbidity risk [24]. In recent years, more accurate anthropometric indices of obesity have been established, such as conicity index (C-index) and body roundness index (BRI). C-index reflects body fat distribution, with value increasing as abdominal fat accumulates [25]. Compared with BMI and WC, C-index can estimate fat mass in both obese and lean individuals [26], and was found to be significant in estimating type 2 diabetes and cardiovascular disease [25,27]. BRI is a newer anthropometric measure to embody fat distribution, coined by Thomas et al. [28], who established elliptical models based on human body shape to calculate body roundness index. High BRI has been significantly related to increased risk of all-cause mortality, cancer, hypertension, and cardiovascular diseases [29,30,31]. Both indices incorporate height, weight, and waist circumference, offering a more comprehensive reflection of fat distribution than traditional measures. However, most existing studies focus on the association of adiposity indices with single diseases, and relationships between C-index and BRI with multimorbidity remain understudied. Furthermore, it is not known whether C-index and BRI are better at discriminating multimorbidity compared with BMI, WC and WHR.

If C-index and BRI are strongly associated with multimorbidity, they could serve as simple, non-invasive screening tools for multimorbidity risk stratification and help to identify individuals who may need further comprehensive nutritional assessment. Hence, to fill this research gap and to explore the potential utility of these indices for risk stratification in nutritional assessment practice, we aimed to investigate the associations of C-index and BRI with multimorbidity among adults in Guangzhou, China, and to compare their discriminative ability with that of traditional adiposity indices (BMI, WC, and WHR).

## 2. Materials and Methods

### 2.1. Study Participants

This study is based on the Fourth Adult Chronic Disease and Risk Factor Surveillance Program in Guangzhou in 2018. A multi-stage stratified cluster sampling method was adopted to survey permanent residents aged 18 years or above. In the first stage, no fewer than five community health centers or township hospitals were randomly selected from each of Guangzhou’s 11 administrative districts, with the number of selections proportional to the population size of each district. In the second stage, investigators randomly selected two neighborhood or village committees from the coverage area of each sampled community health center or township hospital. In the third stage, 100 to 200 households were randomly selected from the jurisdiction of each sampled neighborhood or village committee. Finally, all individuals aged 18 years and above from the selected households were included as study participants. The inclusion criteria for participants were as follows: (1) permanent residents of Guangzhou, (2) aged 18 years or above, and (3) willing to participate in the study and complete the questionnaire survey and related examinations. Participants were excluded if they (1) had severe mental illnesses that prevented them from cooperating with the study or (2) had missing key variables in the returned questionnaires.

Among the 15,727 valid questionnaires collected in the surveillance program, 195 were excluded due to missing key variables relevant to this study, resulting in a final sample of 15,532 valid questionnaires. The study protocol was approved by the Ethics Committee of the Guangzhou Center for Disease Control and Prevention (GZCDC-ECHR-2021P0001), and all the surveys were conducted after obtaining written informed consent from the participants. The questionnaire used for data collection is provided in Supplementary Material S1.

### 2.2. Basic Characteristics

Standardized questionnaire surveys and physical examinations were employed. The questionnaire was designed to collect comprehensive data on the participants’ sociodemographic characteristics (including sex, age, place of residence, educational level, marital status, and occupation), lifestyle factors (including smoking, alcohol consumption, and physical activity patterns), and overall health status. Physical examinations encompassed standardized measurements of height, body weight, waist circumference (WC), and hip circumference.

Height and body weight were measured using a calibrated electronic stadiometer and scale under rigorously standardized protocols. The participants were instructed to fast and remove their footwear, ensuring that their heels, buttocks, and head were in full contact with the vertical board to maximize measurement precision. Waist circumference was measured with the participants in an upright position, using a non-elastic measuring tape placed horizontally at the midpoint between the iliac crest and the lower margin of the rib cage. The Global Physical Activity Questionnaire (GPAQ) was employed to evaluate physical activity levels among the participants [32]. This instrument comprises 16 items assessing various domains of physical activity, including occupational activities, household chores, transportation-related activities, and leisure-time exercise. The participants reported the duration and intensity of their physical activities based on predefined response options. In accordance with the World Health Organization’s guidelines for physical activity in adults, the participants were classified as having sufficient physical activity if they met any of the following criteria: (1) engaging in at least 150 min of moderate-intensity physical activity per week, (2) performing at least 75 min of vigorous-intensity physical activity per week, or (3) achieving a combined total energy expenditure of ≥600 MET-min (metabolic equivalent minutes) per week through a combination of moderate- and vigorous-intensity activities [33]. The Cronbach’s α coefficient of the GPAQ in this study was 0.79.

### 2.3. Conicity Index and Body Roundness Index

C-index and BRI are indices mainly calculated by fixed formulas based on anthropometric indices, including individual weight, height and waist circumference. The formulas are as follows [25,28]:

$$C-Index=\frac{WC(m)}{0.109\times \sqrt{\frac{weight(kg)}{height(m)}}}$$

$$BRI=364.2-365.5\times \sqrt{1-{\left[\frac{WC(cm)/2\pi }{0.5\times height(cm)}\right]}^{2}}$$

The C-index and BRI were each divided into quartiles (Q1–Q4). Sample-specific quartiles were used to reflect the distribution of these indices in our study population, as no universally accepted clinical cut-off values are currently available for C-index and BRI. This approach allows a practical risk stratification based on relative rank within the population.

In this study, the quartiles of C-index were as follows: Q1, C-Index < 1.167; Q2, 1.167 ≤ C-Index < 1.226; Q3, 1.226 ≤ C-Index < 1.285; and Q4, C-Index ≥ 1.285. Similarly, for BRI, the quartiles were: Q1, BRI < 2.832; Q2, 2.832 ≤ BRI < 3.607; Q3, 3.607 ≤ BRI < 4.428; and Q4, BRI ≥ 4.428.

### 2.4. Definition of Multimorbidity

Multimorbidity was defined as the co-occurrence of two or more chronic conditions within an individual. A total of 12 types of chronic diseases were included in the scope of this study on multimorbidity, including hypertension, diabetes, dyslipidemia, coronary heart disease, stroke, asthma, chronic obstructive pulmonary disease, chronic digestive system diseases, chronic urinary system diseases, musculoskeletal diseases, neck and low back diseases, and malignant tumors. Disease ascertainment followed the standardized workflow of the Fourth Adult Chronic Disease and Risk Factor Surveillance Program of Guangzhou. For hypertension, diabetes, and dyslipidemia, diagnoses were ascertained through either (1) on-site physical and laboratory measurements, supplemented with health archive records (including medical history and medication history), or (2) self-reported physician diagnosis. The remaining nine disease conditions were identified solely by self-reported physician diagnosis.

### 2.5. Quality Control

Monitoring plan, on-site investigation plan, implementation manual, questionnaire, and quality control plan were formulated before the launch of this study. This study adopted a multi-stage random sampling method to ensure the representativeness of the population. Internal pre-investigation was conducted to ensure the scientificity and practicality, and leading questions were avoided. Standard investigation guidelines and operation procedures were formulated, and all the investigators were given unified training to ensure the consistency of the data collection process. Each site has a quality controller who conducts real-time quality control of the investigation process and corrects errors to ensure the integrity and logicality of the data.

### 2.6. Statistical Methods

R (version 4.4.1, R Foundation for Statistical Computing, Vienna, Austria) was used for data analysis. Both C-index and BRI were divided into quartiles (Q1–Q4). Count data were described using [*n* (%)], and the chi-square test was used to compare categorical variables between groups. Baseline features were described as numbers (percentages) for categorical variables, and group comparisons were performed using chi-squared tests. A logistic regression model was established to estimate the odds ratio (OR) and 95% confidence interval (CI) to evaluate the association between the C-index, BRI and multimorbidity. Three sets of models were constructed in this study: an unadjusted model (Model 1), a model adjusted for age and gender based on Model 1 (Model 2), and a fully adjusted model that included place of residence, educational level, marital status, occupation, smoking, alcohol drinking, and physical activity based on Model 2 (Model 3). Restricted cubic spline (RCS) was employed to explore the nonlinear dose–response relationship between the C-index, BRI and multimorbidity. Additionally, the receiver operating characteristic (ROC) curve analysis with DeLong’s test was performed to analyze the discriminative ability of different obesity indices in identifying individuals with multimorbidity, and the area under the curve (AUC) was calculated. And *p* < 0.05 was considered to indicate a statistically significant.

## 3. Results

### 3.1. Baseline Characteristics

Table 1 presents the baseline characteristics of the study participants. A total of 15,532 subjects were included, among whom 6050 (38.95%) had multimorbidity. Among the subjects, 9367 (60.31%) were female, and the majority were aged 45–54 years (21.46%) and 55–64 years (25.55%). There were statistically significant differences in the prevalence of multimorbidity among the participants with different genders, ages, places of residence, educational levels, marital statuses, occupations, smoking statuses, alcohol drinking statuses, physical activity statuses, C-index and BRI (*p* < 0.05).

Table 2 presents the baseline characteristics among the participants in different quartiles of C-index and BRI. In this study, the median of the C-index was 1.226, with an interquartile range of (1.167, 1.285). According to the quartile division, there were 3888 people (25.03%) in Q1, 3865 people (24.88%) in Q2, 3885 people (25.01%) in Q3, and 3894 people (25.07%) in Q4. Additionally, the median of the BRI was 3.607, with an interquartile range of (2.832, 4.428). There were 3883 people (25.00%) in Q1, 3882 people (24.99%) in Q2, 3883 people (25.00%) in Q3, and 3884 people (25.01%) in Q4. This study’s results revealed significant differences in gender, age, places of residence, educational level, marital status, occupations, smoking status, alcohol drinking status, and physical activity status among different C-index groups and BRI groups (*p* < 0.05).

### 3.2. Association of C-Index and BRI with Multimorbidity

As presented in Table 3, logistic regression analysis was conducted to examine the associations of C-index and BRI with the risk of multimorbidity. Gender, age, place of residence, educational level, marital status, occupation, smoking status, alcohol drinking status, and physical activity status were included to construct a multivariate logistic regression model. In the fully adjusted model, the participants in C-index Q2, Q3 and Q4 showed a higher risk of multimorbidity, with ORs of 1.25 (95% CI: 1.12–1.39), 1.70 (95% CI: 1.53–1.90), and 1.98 (95% CI: 1.78–2.21), respectively, when compared to C-index Q1. Similarly, after adjusting for covariates, the ORs for BRI Q2, Q3 and Q4 compared to BRI Q1 were 1.30 (95% CI: 1.16–1.45), 1.88 (95% CI: 1.69–2.10) and 3.05 (95% CI: 2.74–3.41). This study revealed a higher risk in higher quartiles of C-index and BRI, a finding that was consistent and robust across both the crude model (Model 1) and the adjusted models (Model 2, 3). The association between C-index, BRI and the risk of multimorbidity exhibited a strong linear trend (*p*-trend < 0.001).

**Figure 1:**
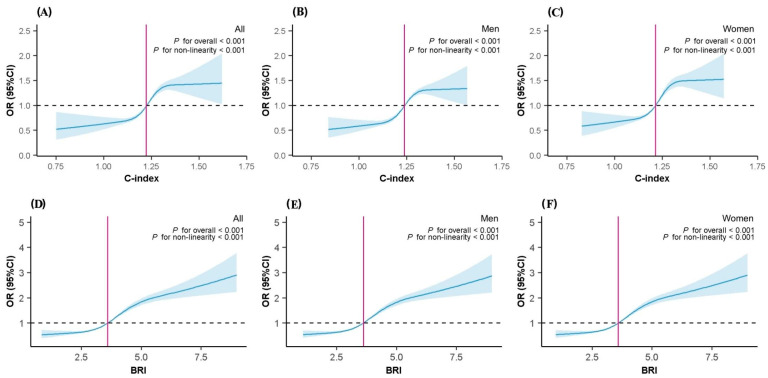
Relationship of conicity index (C-index) and body roundness index (BRI) with the risk of multimorbidity for all the participants and for subgroups of men and women. Note: OR: odds ratio; 95% CI: 95% confidence interval; C-index: conicity index; BRI: body roundness index. (**A**) Dose–response relationship between C-index and risk of multimorbidity in all the participants. (**B**) Dose–response relationship between C-index and risk of multimorbidity in men. (**C**) Dose–response relationship between C-index and risk of multimorbidity in women. (**D**) Dose–response relationship between BRI and risk of multimorbidity in all the participants. (**E**) Dose–response relationship between BRI and risk of multimorbidity in men. (**F**) Dose–response relationship between BRI and risk of multimorbidity in women.

Restricted cubic spline analysis showed a significant dose–response relationship between C-index, BRI and multimorbidity, as shown in Figure 1. The results indicate that the risk of multimorbidity increased with higher levels of C-index and BRI. The associations of C-index and BRI with multimorbidity were linear in all the participants and in subgroups of sex.

### 3.3. Receiver Operating Characteristic Curve Analysis

**Figure 2:**
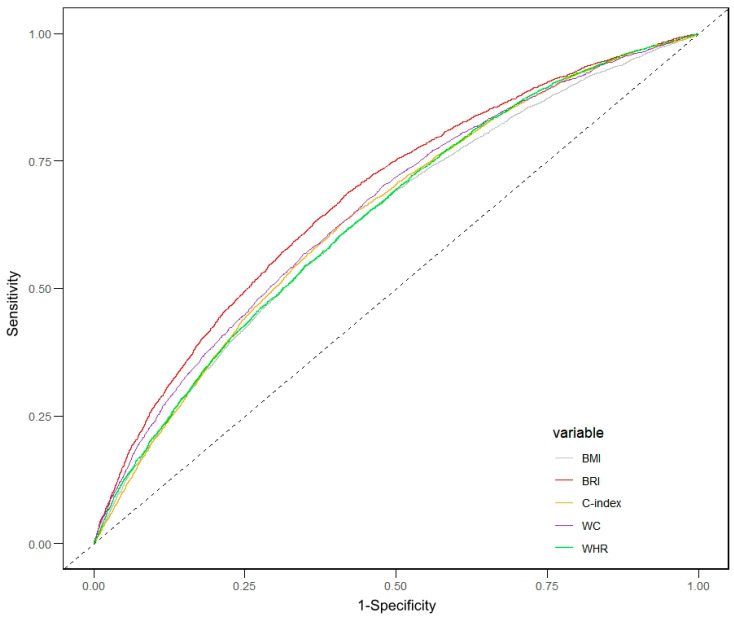
Receiver operating characteristic curve analysis of different adiposity indices in discriminating multimorbidity. Note: BMI: body mass index; BRI: body roundness index; C-index: conicity index; WC: waist circumference; WHR: waist-to-hip ratio.

The discriminative abilities of five adiposity indices for multimorbidity are presented in Figure 2. The areas under the receiver operating characteristic curve (AUC) were 0.6316 (95% CI: 0.6227–0.6405) for BMI, 0.6782 (95% CI: 0.6697–0.6868) for BRI, 0.6427 (95% CI: 0.6339–0.6515) for C-index, 0.6537 (95% CI: 0.6450–0.6625) for WC, and 0.6403 (95% CI: 0.6315–0.6491) for WHR. Pairwise DeLong’s tests were performed to compare differences in AUC between indices, with Bonferroni correction applied for 10 pairwise comparisons. The full statistical results are provided in Supplementary Table S1. After correction, BRI had a statistically significantly higher AUC than all the other four indices (all adjusted *p* < 0.001). WC also had a significantly higher AUC than C-index, BMI and WHR (both adjusted *p* < 0.01). No statistically significant differences were found between C-index and BMI, between C-index and WHR, or between BMI and WHR after Bonferroni correction. Although BRI’s overall ability to distinguish multimorbidity was modest (AUC < 0.7), it showed the highest relative discriminative performance among the evaluated anthropometric indices. Therefore, BRI and other adiposity indices should serve as auxiliary indicators for preliminary risk screening but not as standalone diagnostic tools for multimorbidity.

## 4. Discussion

### 4.1. Main Findings

This study, leveraging data from Guangzhou’s Fourth Adult Chronic Disease and Risk Factor Surveillance Program, sought to examine the associations of two adiposity indices, including the conicity index (C-index) and body roundness index (BRI), with the risk of multimorbidity, and to examine the implications in community-based nutritional risk stratification. Our findings revealed a multimorbidity prevalence of 38.95% among Guangzhou adults. The results of logistic regression analysis and restricted cubic spline (RCS) curve analysis showed significant dose-dependent relationships between C-index, BRI and multimorbidity. These associations remained significant even after adjusting for various risk factors such as gender, age, place of residence, educational level, marital status, occupation, smoking status, alcohol drinking status, and physical activity status. Additional confidence in these results was obtained through subgroup RCS analysis, with each incremental increase in C-index and BRI corresponding to elevated multimorbidity risk. Receiver operating characteristic (ROC) curve analysis further indicated that BRI demonstrated better discriminative ability for multimorbidity compared to conventional anthropometric measures, whereas the ability of C-index was relatively lower. These findings provide preliminary evidence for multimorbidity risk stratification and may offer reference for subsequent nutritional assessment practice.

### 4.2. Comparison with Previous Studies

The multimorbidity prevalence observed in this study (38.95%) was comparable to the 37.2%, which was reported as the overall global prevalence of multimorbidity by a meta-analysis [2]. Additionally, we found that older adults, men, urban living participants faced a greater multimorbidity burden, in line with previous studies [3]. Interestingly, this study showed a discrepancy from prior reports that higher multimorbidity prevalence was found among former smokers and physically active participants [34,35]. This discrepancy is likely attributable to reverse causality inherent to the cross-sectional design: individuals diagnosed with chronic conditions often modify their lifestyles after diagnosis, such as quitting smoking and increasing physical activity on medical advice. Therefore, reverse causality should be considered in the cross-sectional measurement as it cannot distinguish whether the health behavior preceded the disease or was adopted after disease onset.

### 4.3. Potential Biological Mechanisms

Previous studies have demonstrated a close relationship between abdominal obesity and multiple chronic diseases. Obesity and abdominal obesity elevate the risk of hypertension, diabetes, kidney cancer, and cardiovascular disease [36,37,38,39]. Mechanistically, abdominal adiposity promotes macrophage infiltration into adipose tissue, triggering the production of pro-inflammatory cytokines (e.g., interleukin-6, tumor necrosis factor-α). This leads to oxidative stress, endothelial dysfunction, and atherosclerosis, thereby increasing cardiovascular diseases [40,41]. Furthermore, visceral fat accumulation disrupts adipokine profiles (e.g., adiponectin, leptin), promoting chronic inflammation and cellular metabolic dysfunction [42]. Increased abdominal visceral fat is also associated with increased insulin resistance and dyslipidemia, further affecting the function of the renal sodium metabolism system and sympathetic nervous system, inducing or aggravating chronic diseases such as hyperinsulinemia, hypertension, diabetes, and nephropathy [43,44,45]. Collectively, these pathways highlight abdominal adiposity as a unifying risk factor for multiple chronic diseases and multimorbidity.

### 4.4. Performance of C-Index and BRI in Discriminating Multimorbidity

China faces a significant obesity epidemic, with over 35% of adults presenting with abdominal obesity [46]. Even though it was known that abdominal adiposity could be accurately detected by radiological imaging techniques, such as dual-energy X-ray absorptiometry (DEXA), magnetic resonance imaging (MRI), computed tomography (CT), and dual bioelectrical impedance analysis (BIA) [47,48], these methods are not widely accepted by the public nor easily applied in routine clinical practices on account of complex operations, high costs, radiation exposure hazards [49,50]. Assessment based on anthropometric measurements is less precise and dependent on trained personnel. However, they are more feasible for large-scale screening, where simplicity and low cost are prioritized over maximal precision. The conicity index (C-index) and body roundness index (BRI) are adiposity indices derived from geometric models incorporating anthropometric parameters including height, weight, and waist circumference, mainly used to assess body fat distribution, especially the accumulation of abdominal adiposity [29,51,52]. These indices are designed to assess abdominal obesity through geometric modeling, avoiding radiation exposure, and enable direct comparisons of abdominal obesity between individuals or populations [31,53]. Therefore, though their discriminative accuracy is limited by the modest AUC in ROC analysis, C-index and BRI offer advantages over traditional methods in reflecting abdominal fat distribution, and hold potential for large-scale preliminary risk screening research of chronic diseases and multimorbidity.

C-index is commonly utilized to evaluate abdominal visceral fat and has been identified as a marker of body fat distribution, with its value rising as abdominal fat accumulates [25]. It was demonstrated as a superior anthropometric indicator in identifying the distribution of body fat, particularly abdominal obesity, compared to BMI [52]. Our study found significant associations between C-index and multimorbidity by multivariate logistic regression analysis, showing that higher C-index quartiles (Q2–Q4) were associated with progressively increased multimorbidity risk, compared to the lower C-index quartile (Q1), which was consistent with previous studies. For instance, a cross-sectional study among Mexican populations reported C-index was a better predictor of comprehensive adiposity-based chronic diseases [54], and a study of Brazilian women aged 20–59 years found that the C-index was an important tool in estimating the risk of diabetes, hypertension and dyslipidemia [55].

Growing evidence claimed the body roundness index (BRI) was superior to traditional indicators such as BMI, WC, and hip circumference (HC) in predicting body fat distribution and visceral fat percentage [56]. Similar to C-index, our study demonstrated a significant positive association between BRI and risk of multimorbidity among individuals aged 18 years and above, with higher quartiles conferring elevated risks even after adjusting for confounding variables. This finding was in line with a previous study, which revealed high BRI had a markedly increased risk of developing cardiometabolic multimorbidity [56].

Restricted cubic spline (RCS) curve analysis revealed significant dose–response relationships between C-index, BRI and risk of multimorbidity, with risk increasing linearly as indices rose above threshold values: C-index > 1.223 or BRI > 3.598. Sex-stratified analysis demonstrated consistent trends, though cut-off points differed slightly (C-index: 1.238 in males vs. 1.214 in females; BRI: 3.622 in males vs. 3.594 in females). These findings highlight the need to pay greater attention to multimorbidity risk among women, even at lower adiposity index values compared to men. This novel observation may inspire future research on adiposity indices and inform targeted prevention strategies. Receiver operating characteristic (ROC) curve analysis further validated that BRI had a statistically significantly better discriminative performance for multimorbidity (AUC = 0.678) than traditional measures including WHR, BMI, and WC, though the overall discriminative ability remained modest. However, contrary to our finding, a previous study advocated that waist circumference (WC) was more strongly correlated with the risk of multimorbidity [57]. This discrepancy may be attributed to variations in geographical location, lifestyle and age composition of study populations. Future studies should focus on refining risk stratification models by integrating additional biomarkers and longitudinal data to improve discriminative accuracy.

### 4.5. Implications for Nutritional Step-Care in Multimorbidity

Stepped care models have gained increasing attention as a promising framework for chronic disease management [58]. Beyond the strong statistical associations between the adiposity indices and multimorbidity, our findings provide preliminary evidence to support community-based risk stratification. The dose–response relationship and relatively discriminative performance of BRI (AUC = 0.678) support using these indices as simple, low-cost preliminary screening tools to identify individuals at higher multimorbidity risk who warrant further clinical and nutritional assessment. If validated, these indices might eventually be integrated into a stepped nutritional care model: low-risk individuals receive general nutritional advice, while high-risk individuals, for instance those in the top BRI quartile, receive more detailed nutritional evaluation and support in primary care settings, potentially including referral to a dietitian for nutritional counseling. Importantly, both indices are non-invasive and require only basic anthropometric measurements, which may facilitate their use in large-scale community settings.

It is important to emphasize that data from this cross-sectional study do not support direct causal claims about nutritional interventions. Rather, they generate hypotheses for future prospective studies to verify whether adiposity-index-guided nutritional management can improve health outcomes in people with or at risk of multimorbidity.

### 4.6. Strengths and Limitations

Some strengths should be introduced in this study. Firstly, conducted within a representative surveillance program in Guangzhou, this study provides a large sample size and wide coverage, and robust evidence linking C-index and BRI to multimorbidity in Chinese adults, offering critical data for supporting community-based risk stratification and helping identify individuals who warrant further clinical and nutritional assessment. Secondly, while previous studies have primarily focused on the relationship between abdominal obesity indices and single chronic disease, we utilized both C-index and BRI as innovative anthropometric indicators, offering a simple and accurate method for assessing the risk of multimorbidity. Thirdly, in this study, we compared the discriminative abilities for multimorbidity of these new abdominal indices with those of traditional indicators.

However, several potential limitations should be acknowledged. First, the cross-sectional design could not determine the causal relationship between C-index, BRI and multimorbidity. Reverse causality is plausible as individuals with multiple chronic conditions may change their smoking, alcohol consumption, and physical activity levels after diagnosis, which could in turn affect adiposity index values. Second, disease ascertainment of hypertension, diabetes, and dyslipidemia was verified through multi-source data including on-site measurements, health archive records and self-reported medical history, with relatively low misclassification risk. However, the remaining nine chronic conditions were ascertained solely by self-reported diagnosis, which may introduce recall bias and differential misclassification. Third, this study focused on dichotomized physical activity level as the core lifestyle covariate and did not incorporate sedentary time in analysis, which may introduce residual confounding. More detailed investigation of sedentary behavior and refined physical activity stratification is warranted in future studies. Moreover, several important potential confounders were not available for analysis due to the predefined scope of the program, including household income and broader socioeconomic indicators, medication use, menopausal status in women, family history, sleep duration, healthcare access, and disease duration. In particular, although we discuss the potential value of C-index and BRI for informing further clinical and nutritional assessment, this study did not collect data on dietary intake or nutritional status. It is unknown whether interventions would improve nutritional status and clinical outcomes in multimorbidity. Future prospective studies are needed to establish causality and to test the effectiveness of nutritional interventions guided by these indices.

## 5. Conclusions

In conclusion, this study identifies high multimorbidity prevalence among adults in Guangzhou, and indicates significant, dose-dependent positive correlations between the two abdominal adiposity indices C-index and BRI, and multimorbidity risk. Among the indices evaluated, BRI showed relatively superior discriminative ability compared with traditional adiposity indices. C-index and BRI are simple, non-invasive indices. Thus, in community-based settings, incorporating these indices into risk-stratification workflows and step-care nutritional assessment may help identify individuals with high risk of multimorbidity and those who warrant further clinical and nutritional evaluation. These findings are preliminary and hypothesis-generating. Further prospective studies and interventional research are required to verify their clinical utility and inform evidence-based nutritional care pathways for multimorbidity management.

## Figures and Tables

**Table 1:** Comparison of basic characteristics and prevalence of multimorbidity.

Variables	Total	Multimorbidity [*n* (%)]	*χ* ^2^	*p* Value
Sex			22.525	<0.001
Men	6165	2543 (42.03)		
Women	9367	3507 (57.97)		
Age groups (years)			2528.533	<0.001
18–34	2791	266 (4.40)		
35–44	2632	523 (8.64)		
45–54	3333	1315 (21.74)		
55–64	3969	2172 (35.90)		
≥65	2807	1774 (29.32)		
Location			8.411	0.004
Urban	11,015	4210 (69.59)		
Rural	4517	1840 (30.41)		
Education			659.406	<0.001
Primary school or below	3837	2011 (33.24)		
Middle school	4455	1794 (29.65)		
High school	3879	1474 (24.36)		
College or further	3361	771 (12.74)		
Marital status			474.960	<0.001
Unmarried	1077	132 (2.18)		
Married	13,516	5370 (88.76)		
Divorce/Widowed	939	548 (9.06)		
Occupation			604.436	<0.001
Physical worker	7166	2435 (40.25)		
Brain worker	2673	706 (11.67)		
Retired/Inoccupation	5693	2909 (48.08)		
Smoking			202.405	<0.001
Non-smoker	12,076	4441 (73.40)		
Ex-smoker	808	494 (8.17)		
Smoker	2648	1115 (18.43)		
Alcohol drinking			10.508	0.001
No	10,968	4182 (69.12)		
Yes	4564	1868 (30.88)		
Sufficient physical exercise			138.394	<0.001
No	3777	1164 (19.24)		
Yes	11,755	4886 (80.76)		
C-index			859.282	<0.001
Q1	3888	908 (15.01)		
Q2	3865	1310 (21.65)		
Q3	3885	1731 (28.61)		
Q4	3894	2101 (34.73)		
BRI			1304.685	<0.001
Q1	3883	828 (13.69)		
Q2	3882	1226 (20.26)		
Q3	3883	1695 (28.02)		
Q4	3884	2301 (38.03)		

Note: C-index: conicity index; BRI: body roundness index. Percentages in the “Multimorbidity [n (%)]” column represent the proportion of each subgroup within the total multimorbid population.

**Table 2:** Comparison of baseline characteristics among participants in different quartiles of conicity index and body roundness index.

Variables	C-Index						BRI					
	Q1 (*n* = 3888)	Q2 (*n* = 3865)	Q3 (*n* = 3885)	Q4 (*n* = 3894)	*χ* ^2^	*p*	Q1 (*n* = 3883)	Q2 (*n* = 3882)	Q3 (*n* = 3883)	Q4 (*n* = 3884)	*χ* ^2^	*p*
Sex					327.750	<0.001					64.783	<0.001
Men	1085 (27.91)	1564 (40.47)	1791 (46.10)	1725 (44.30)			1446 (37.24)	1610 (41.47)	1706 (43.94)	1403 (36.12)		
Women	2803 (72.09)	2301 (59.53)	2094 (53.90)	2169 (55.70)			2437 (62.76)	2272 (58.53)	2177 (56.06)	2481 (63.88)		
Age groups (years)					2076.960	<0.001					2073.151	<0.001
18–34	1273 (32.74)	722 (18.68)	492 (12.66)	304 (7.81)			1392 (35.85)	704 (18.13)	437 (11.25)	258 (6.64)		
35–44	914 (23.51)	769 (19.90)	596 (15.34)	353 (9.07)			826 (21.27)	805 (20.74)	625 (16.10)	376 (9.68)		
45–54	757 (19.47)	897 (23.21)	963 (24.79)	716 (18.39)			645 (16.61)	900 (23.18)	973 (25.06)	815 (20.98)		
55–64	634 (16.31)	992 (25.67)	1131 (29.11)	1212 (31.12)			653 (16.82)	955 (24.60)	1108 (28.53)	1253 (32.26)		
≥65	310 (7.97)	485 (12.55)	703 (18.10)	1309 (33.62)			367 (9.45)	518 (13.34)	740 (19.06)	1182 (30.43)		
Location					15.332	0.002					73.834	<0.001
Urban	2836 (72.94)	2751 (71.18)	2743 (70.60)	2685 (68.95)			2799 (72.08)	2918 (75.17)	2711 (69.82)	2587 (66.61)		
Rural	1052 (27.06)	1114 (28.82)	1142 (29.40)	1209 (31.05)			1084 (27.92)	964 (24.83)	1172 (30.18)	1297 (33.39)		
Education					800.980	<0.001					1185.796	<0.001
Primary school or below	602 (15.48)	826 (21.37)	962 (24.76)	1447 (37.16)			568 (14.63)	755 (19.45)	998 (25.70)	1516 (39.03)		
Middle school	1024 (26.34)	1117 (28.90)	1196 (30.79)	1118 (28.71)			947 (24.39)	1093 (28.16)	1214 (31.26)	1201 (30.92)		
High school	1005 (25.85)	1042 (26.96)	1010 (26.00)	822 (21.11)			1036 (26.68)	1098 (28.28)	988 (25.44)	757 (19.49)		
College or further	1257 (32.33)	880 (22.77)	717 (18.46)	507 (13.02)			1332 (34.30)	936 (24.11)	683 (17.59)	410 (10.56)		
Marital status					453.882	<0.001					743.272	<0.001
Unmarried	504 (12.96)	243 (6.29)	198 (5.10)	132 (3.39)			598 (15.40)	233 (6.00)	142 (3.66)	104 (2.68)		
Married	3234 (83.18)	3443 (89.08)	3468 (89.27)	3371 (86.57)			3143 (80.94)	3473 (89.46)	3508 (90.34)	3392 (87.33)		
Divorce/Widowed	150 (3.86)	179 (4.63)	219 (5.64)	391 (10.04)			142 (3.66)	176 (4.53)	233 (6.00)	388 (9.99)		
Occupation					311.437	<0.001					471.783	<0.001
Physical worker	1870 (48.10)	1811 (46.86)	1842 (47.41)	1643 (42.19)			1857 (47.82)	1771 (45.62)	1832 (47.18)	1706 (43.92)		
Brain worker	879 (22.61)	728 (18.84)	599 (15.42)	467 (11.99)			941 (24.23)	779 (20.07)	582 (14.99)	371 (9.55)		
Retired/Inoccupation	1139 (29.30)	1326 (34.31)	1444 (37.17)	1784 (45.81)			1085 (27.94)	1332 (34.31)	1469 (37.83)	1807 (46.52)		
Smoking					253.881	<0.001					51.657	<0.001
Non-smoker	3317 (85.31)	3071 (79.46)	2833 (72.92)	2855 (73.32)			3096 (79.73)	3032 (78.10)	2924 (75.30)	3024 (77.86)		
Ex-smoker	102 (2.62)	179 (4.63)	236 (6.07)	291 (7.47)			138 (3.55)	187 (4.82)	236 (6.08)	247 (6.36)		
Smoker	469 (12.06)	615 (15.91)	816 (21.00)	748 (19.21)			649 (16.71)	663 (17.08)	723 (18.62)	613 (15.78)		
Alcohol drinking					27.429	<0.001					45.017	<0.001
No	2774 (71.35)	2688 (69.55)	2651 (68.24)	2855 (73.32)			2681 (69.04)	2672 (68.83)	2709 (69.77)	2906 (74.82)		
Yes	1114 (28.65)	1177 (30.45)	1234 (31.76)	1039 (26.68)			1202 (30.96)	1210 (31.17)	1174 (30.23)	978 (25.18)		
Sufficient physical exercise					11.960	0.0075					21.867	<0.001
No	952 (24.49)	890 (23.03)	916 (23.58)	1019 (26.17)			1014 (26.11)	994 (25.61)	861 (22.17)	908 (23.38)		
Yes	2936 (75.51)	2975 (76.97)	2969 (76.42)	2875 (73.83)			2869 (73.89)	2888 (74.39)	3022 (77.83)	2976 (76.62)		

Note: C-index: conicity index; BRI: body roundness index.

**Table 3:** Multiple logistic regression analysis of conicity index and body roundness index on the risk of multimorbidity.

	Model 1 ^a^		Model 2 ^b^		Model 3 ^c^	
	OR (95% CI)	*p*	OR (95% CI)	*p*	OR (95% CI)	*p*
C-index						
Q1	1	-	1	-	1	-
Q2	1.68 (1.55, 1.86)	<0.001	1.26 (1.13, 1.40)	<0.001	1.25 (1.12, 1.39)	<0.001
Q3	2.64 (2.39, 2.91)	<0.001	1.71 (1.53, 1.90)	<0.001	1.70 (1.53, 1.90)	<0.001
Q4	3.85 (3.49, 4.24)	<0.001	1.97 (1.77, 2.19)	<0.001	1.98 (1.78, 2.21)	<0.001
Ptrend		<0.001		<0.001		<0.001
BRI						
Q1	1	-	1	-	1	-
Q2	1.70 (1.54, 1.89)	<0.001	1.30 (1.16, 1.45)	<0.001	1.30 (1.16, 1.45)	<0.001
Q3	2.86 (2.59, 3.16)	<0.001	1.88 (1.69, 2.11)	<0.001	1.88 (1.69, 2.10)	<0.001
Q4	5.36 (4.85, 5.93)	<0.001	3.02 (2.71, 3.37)	<0.001	3.05 (2.74, 3.41)	<0.001
Ptrend		<0.001		<0.001		<0.001

Note: OR: odds ratio; 95% CI: 95% confidence interval; C-index: conicity index; BRI: body roundness index. ^a^ Model 1: Crude model. ^b^ Model 2: Adjusted for gender and age. ^c^ Model 3: Adjusted for gender, age, place of residence, educational level, marital status, occupation, smoking status, alcohol drinking status, and physical activity status.

## Data Availability

The data that support the findings are available upon request from the corresponding author. The data are not publicly available due to privacy or ethical restrictions.

## Supplementary Materials

The following supporting information can be downloaded at https://www.mdpi.com/article/10.3390/nu18142286/s1: Supplementary Material S1: Questionnaire; Table S1: Pairwise comparisons of AUCs between adiposity indices using DeLong’s test with Bonferroni correction.

## Abbreviations

The following abbreviations are used in this manuscript:

C-index	Conicity index
BRI	Body roundness index
BMI	Body mass index
WC	Waist circumference
WHR	Waist-to-hip ratio
HC	Hip circumference
GPAQ	The Global Physical Activity Questionnaire
DEXA	Dual-energy X-ray absorptiometry
MRI	Magnetic resonance imaging
CT	Computed tomography
BIA	Bioelectrical impedance analysis
