# Supplementary material for: Association of Conicity Index and Body Roundness Index with Multimorbidity Among Adults in Guangzhou, China: A Cross-Sectional Study and Implications for Nutritional Risk Stratification
Source: Nutrients. 2026 Jul 13;18(14):2286. doi: 10.3390/nu18142286 (PMC13414857; doi:10.3390/nu18142286)
Supplement: Supplementary file 1 [file nutrients-18-02286-s001.zip › Supplementary material S1-Questionnaire.pdf]

Supplementary material S1

Questionnaire

ID: ________________
Participant phone number: ________________
Date:________________
Researcher:________________

Dear Resident,
We are conducting a research study to explore the association between C-index, BRI and multimorbidity among adults in Guangzhou. Your participation is crucial as it will not only help you gain insights into your own health but also contribute to improving health management strategies for the entire city. Additionally, your valuable input can drive advancements in medical research on obesity - related diseases. All participation is voluntary, and all information collected will be kept strictly confidential. By filling out this questionnaire, you are believed to consent to participate in our project. Thank you for your time and contribution.

Part A: Basic information

A1 Your age: ______ years old

A2 Your gender:

1) Men 2) Women

A3 Your location:

1) Urban 2) Rural

A4 Your educational level:

1) Primary school or below 2) Middle school
3) High school 4) College or further

A5 Your marital status:

1) Unmarried 2) Married
3) Divorced/Widowed

A6 Your occupation:

1) Physical worker 2) Brain worker
3) Retired/ Inoccupation

Part B: Lifestyle factors

Please select your current status.

B1 Cigarette smoking:

1) Non-smoker 2) Ex-smoker
3) Smoker

B2 Alcohol drinking :

1) Yes 2) No

Part C: Physical exercise

Next I am going to ask you about the time you spend doing different types of physical activity in a typical week. Please answer these questions even if you do not consider yourself to be a physically active person.

Think first about the time you spend doing work. Think of work as the things that you have to do such as paid or unpaid work, study/training, household chores, harvesting food/crops, fishing or hunting for food, seeking employment. [Insert other examples if needed]. In answering the following questions 'vigorous-intensity activities' are activities that require hard physical effort and cause large increases in breathing or heart rate, 'moderate-intensity activities' are activities that require moderate physical effort and cause small increases in breathing or heart rate.

Questions		Response	Code
**Activity at work**			
1	Does your work involve vigorous-intensity activity that causes large increases in breathing or heart rate like *[carrying or lifting heavy loads, digging or construction work*] for at least 10 minutes continuously?<br>*[INSERT EXAMPLES] (USE SHOWCARD)*	Yes 1<br>No 2 *If No, go to P 4*	P1
2	In a typical week, on how many days do you do vigorous-intensity activities as part of your work?	Number of days └─┘	P2
3	How much time do you spend doing vigorous-intensity activities at work on a typical day?	Hours : minutes └─┴─┘ : └─┴─┘<br>hrs mins	P3 (a-b)
4	Does your work involve moderate-intensity activity that causes small increases in breathing or heart rate such as brisk walking *[or carrying light loads*] for at least 10 minutes continuously?<br>*[INSERT EXAMPLES] (USE SHOWCARD)*	Yes 1<br>No 2 *If No, go to P 7*	P4
5	In a typical week, on how many days do you do moderate-intensity activities as part of your work?	Number of days └─┘	P5
6	How much time do you spend doing moderate-intensity activities at work on a typical day?	Hours : minutes └─┴─┘ : └─┴─┘<br>hrs mins	P6 (a-b)
**Travel to and from places**			
The next questions exclude the physical activities at work that you have already mentioned.<br>Now I would like to ask you about the usual way you travel to and from places. For example to work, for shopping, to market, to place of worship. [insert other examples if needed]			
7	Do you walk or use a bicycle (*pedal cycle*) for at least 10 minutes continuously to get to and from places?	Yes 1<br>No 2 *If No, go to P 10*	P7
8	In a typical week, on how many days do you walk or bicycle for at least 10 minutes continuously to get to and from places?	Number of days └─┘	P8
9	How much time do you spend walking or bicycling for travel on a typical day?	Hours : minutes └─┴─┘ : └─┴─┘<br>hrs mins	P9 (a-b)
**Recreational activities**			
The next questions exclude the work and transport activities that you have already mentioned.<br>Now I would like to ask you about sports, fitness and recreational activities (leisure), [insert relevant terms].			
10	Do you do any vigorous-intensity sports, fitness or recreational (*leisure*) activities that cause large increases in breathing or heart rate like [*running or football,]* for at least 10 minutes continuously?<br>*[INSERT EXAMPLES] (USE SHOWCARD)*	Yes 1<br>No 2 *If No, go to P 13*	P10
11	In a typical week, on how many days do you do vigorous-intensity sports, fitness or recreational (*leisure*) activities?	Number of days └─┘	P11
12	How much time do you spend doing vigorous-intensity sports, fitness or recreational activities on a typical day?	Hours : minutes └─┴─┘ : └─┴─┘<br>hrs mins	P12 (a-b)

**Physical Activity (recreational activities)** contd.			
**Questions**		**Response**	**Code**
13	Do you do any moderate-intensity sports, fitness or recreational (*leisure*) activities that causes a small increase in breathing or heart rate such as brisk walking,(*cycling, swimming, volleyball*)for at least 10 minutes continuously?<br>*[INSERT EXAMPLES] (USE SHOWCARD)*	Yes 1<br>No 2 *If No, go to P16*	P13
14	In a typical week, on how many days do you do moderate-intensity sports, fitness or recreational (*leisure*) activities?	Number of days └┘	P14
15	How much time do you spend doing moderate-intensity sports, fitness or recreational (*leisure*) activities on a typical day?	Hours : minutes └┴┘ : └┴┘ hrs mins	P15 (a-b)
**Sedentary behaviour**			
The following question is about sitting or reclining at work, at home, getting to and from places, or with friends including time spent [sitting at a desk, sitting with friends, travelling in car, bus, train, reading, playing cards or watching television], but do not include time spent sleeping.<br>*[INSERT EXAMPLES] (USE SHOWCARD)*			
16	How much time do you usually spend sitting or reclining on a typical day?	Hours : minutes └┴┘ : └┴┘ hrs min s	P16 (a-b)

Part D:Health status

Have you ever been diagnosed with a certain disease by doctors from primary health centers, community health service centers or medical institutions?

1) None	Yes☐	No☐
2) Hypertension	Yes☐	No☐
3) Diabetes	Yes☐	No☐
4) Dyslipidemia	Yes☐	No☐
5)Coronary heart disease	Yes☐	No☐
6)Stroke	Yes☐	No☐
7)Asthma	Yes☐	No☐
8) Chronic obstructive pulmonary disease	Yes☐	No☐
9) Chronic digestive system diseases	Yes☐	No☐
10) Chronic urinary system diseases	Yes☐	No☐
11)Musculoskeletal diseases	Yes☐	No☐
12) Neck and low back diseases,	Yes☐	No☐
13) Malignant tumors	Yes☐	No☐
14)Other		

Part E: Physical examination (anthropometric measurements)

Next, professional medical staff will use standard medical equipment to measure your height, weight, waist circumference and hip circumference. Thank you for your cooperation.

1) Height:__________cm
2) Weight:__________kg
3) Waist circumference:__________cm
4) hip circumference:__________cm_
