# Supplementary material for: Association of Conicity Index and Body Roundness Index with Multimorbidity Among Adults in Guangzhou, China: A Cross-Sectional Study and Implications for Nutritional Risk Stratification
Source: Nutrients. 2026 Jul 13;18(14):2286. doi: 10.3390/nu18142286 (PMC13414857; doi:10.3390/nu18142286)
Supplement: Supplementary file 1 [file nutrients-18-02286-s001.zip › Supplementary material-Table S1.pdf]

Supplementary material

Table S1. Pairwise comparisons of AUCs between adiposity indices using DeLong's test with Bonferroni correction

Comparison	AUC Difference	95% CI of Difference	Raw P value	Adjusted P value (Bonferroni)
BRI vs C-index	0.036	(0.029,0.042)	< 0.001	< 0.001
BRI vs BMI	0.047	(0.040,0.053)	< 0.001	< 0.001
BRI vs WC	0.025	(0.020,0.029)	< 0.001	< 0.001
BRI vs WHR	0.038	(0.031,0.045)	< 0.001	< 0.001
C-index vs BMI	0.011	(0.000,0.022)	0.0457	0.457
C-index vs WC	-0.011	(-0.017,-0.005)	0.0005	0.005
C-index vs WHR	0.002	(-0.004,0.009)	0.4763	1.000
BMI vs WC	-0.022	(-0.028,-0.016)	< 0.001	< 0.001
BMI vs WHR	-0.009	(-0.018,0.001)	0.0736	0.736
WC vs WHR	0.013	(0.007,0.020)	< 0.001	< 0.001

Note: AUC, area under the receiver operating characteristic curve; CI, confidence interval; BMI, body mass index; BRI, body roundness index; C-index, conicity index; WC, waist circumference; WHR, waist-to-hip ratio. Bonferroni correction was applied for 10 pairwise comparisons.
